# Cotranscriptional and Posttranscriptional Features of the Transcriptome in Soybean Shoot Apex and Leaf

**DOI:** 10.3389/fpls.2021.649634

**Published:** 2021-04-09

**Authors:** Jiafu Zhu, Han Zhao, Fanjiang Kong, Baohui Liu, Min Liu, Zhicheng Dong

**Affiliations:** ^1^Guangzhou Key Laboratory of Crop Gene Editing, Innovative Center of Molecular Genetics and Evolution, School of Life Sciences, Guangzhou Higher Education Mega Center, Guangzhou University, Guangzhou, China; ^2^Provincial Key Laboratory of Agrobiology, Institute of Crop Germplasm and Biotechnology, Jiangsu Academy of Agricultural Sciences, Nanjing, China

**Keywords:** soybean, chromatin-bound RNA, co-transcriptional splicing, non-coding RNA, nascent RNA

## Abstract

Transcription is the first step of central dogma, in which the genetic information stored in DNA is copied into RNA. In addition to mature RNA sequencing (RNA-seq), high-throughput nascent RNA assays have been established and applied to provide detailed transcriptional information. Here, we present the profiling of nascent RNA from trifoliate leaves and shoot apices of soybean. In combination with nascent RNA (chromatin-bound RNA, CB RNA) and RNA-seq, we found that introns were largely spliced cotranscriptionally. Although alternative splicing (AS) was mainly determined at nascent RNA biogenesis, differential AS between the leaf and shoot apex at the mature RNA level did not correlate well with cotranscriptional differential AS. Overall, RNA abundance was moderately correlated between nascent RNA and mature RNA within each tissue, but the fold changes between the leaf and shoot apex were highly correlated. Thousands of novel transcripts (mainly non-coding RNA) were detected by CB RNA-seq, including the overlap of natural antisense RNA with two important genes controlling soybean reproductive development, *FT2a* and *Dt1*. Taken together, we demonstrated the adoption of CB RNA-seq in soybean, which may shed light on gene expression regulation of important agronomic traits in leguminous crops.

## Introduction

Transcription, the first step of gene expression, is accomplished by the multisubunit protein complex RNA polymerase. In eukaryotic cells, RNA polymerase II (RNA Pol II) is involved in protein-coding gene transcription and some non-coding gene transcription. Before maturation, messenger RNA precursors (pre-mRNAs) are subjected to multiple processing steps, including 5′ capping, splicing of introns, 3′ cleavage and polyadenylation, and editing (Bentley, 2014). These steps are known as posttranscriptional processing. However, increasing evidence suggests that most processes are cotranscriptional. For example, introns can be either co- or posttranscriptionally spliced, which is supported by the splicing loops of nascent RNA observed by electron microscopy in *Drosophila melanogaster* and *Chironomus tentans* (Beyer and Osheim, 1988; Baurén and Wieslander, 1994). In addition, high-throughput sequencing of nascent RNA revealed genome-wide cotranscriptional splicing (Khodor et al., 2011; Nojima et al., 2015; Drexler et al., 2020). Studies from budding yeast, flies, and mammals indicated that cotranscriptional splicing frequencies are similarly high, ranging from 75 to 85% (Neugebauer, 2019).

Since Core et al. (2008) published a method wherein the nuclei run on RNA were affinity purified followed by high-throughput sequencing, nascent RNA sequencing (RNA-seq) technologies have significantly improved our ability to analyze transcription at each step across the genome. Rather than steady-state mRNA, nascent RNA-seq detects pre-mRNAs, divergent transcripts, enhancer-derived RNA (eRNA), etc., which are usually unstable and not polyadenylated. Recently, we and another laboratory have reported cotranscriptional splicing in the model plant *Arabidopsis* using genome-wide nascent RNA-seq approaches, plant native elongating transcript sequencing (pNET-seq), and plaNET-seq (Zhu et al., 2018; Kindgren et al., 2020). pNET-seq and plaNET-seq detect nascent RNA through enrichment of transcriptionally engaged RNA Pol II complexes, and splicing intermediates can also be observed when some spliceosomes are copurified with Pol II complexes (Zhu et al., 2018). Moreover, three recent publications directly sequenced the chromatin-bound RNA (CB RNA) of *Arabidopsis* and found genome-wide cotranscriptional splicing (Jia et al., 2020; Li et al., 2020; Zhu et al., 2020). However, the *Arabidopsis* genome is the first plant genome to be sequenced and is compact (140 million base/haploid genome), with an average gene length of 2,000 bp and an average intron length of 180 bp (Arabidopsis Genome Initiative, 2000). While harboring thousands to tens of thousands of genes, plant genome size ranges from approximately 0.1 to 100 gigabases (Pellicer and Leitch, 2020). Therefore, knowledge of transcription obtained from *Arabidopsis* may not be applicable to other plant genomes, especially some complicated crop genomes.

As one of the most important crops, soybean provides protein and oil for humans and livestock. During the past decades, great progress has been made in soybean genome research (Shen et al., 2018; Xie et al., 2019; Liu Y. et al., 2020). Furthermore, many important genes involved in agronomic traits have been characterized *via* genetic, cellular biology, and biochemical approaches (Kasai et al., 2007; Lu et al., 2017, 2020). For example, *Dt1*, which controls soybean growth habits, has been cloned as a *TFL1* homolog encoding a 173-amino-acid peptide (Liu et al., 2010; Tian et al., 2010). *FT2a* and *FT5a*, two distant homologous genes of *Dt1* within the same family, have been shown to play a conserved role in controlling flowering time (Kong et al., 2010; Takeshima et al., 2019; Wu et al., 2017).

Soybean [*Glycine max* (L.) Merr.] is a paleopolyploid derived from two whole genome duplication events approximately 59 and 13 million years ago. It has a relatively complicated and large genome, with a size of approximately 1.1 gigabases (Schmutz et al., 2010). The average gene length is approximately 4,000 bp, and the average intron length is approximately 539 bp in soybean (Shen et al., 2014), which are longer than those in *Arabidopsis*. Despite the considerable transcriptomic analyses of various soybean tissues using mature RNA-seq (Libault et al., 2010; Severin et al., 2010; Shen et al., 2014; Wang et al., 2014; Gazara et al., 2019), genome-wide analysis of nascent RNA from soybean has not yet been reported. In addition to capturing cotranscriptional features, nascent RNA is very sensitive to the detection of unstable regulatory RNAs, such as long non-coding RNAs (ncRNAs). Therefore, the investigation on nascent RNA in soybean would provide a comprehensive description of cotranscriptional characteristics in leguminous crops. Here, we report for the first time the analysis of nascent RNA from the shoot apex and leaf tissues of the soybean cv. Williams 82.

## Results

### Nascent RNA Profiling of Soybean by CB RNA-Seq

**FIGURE 1:**
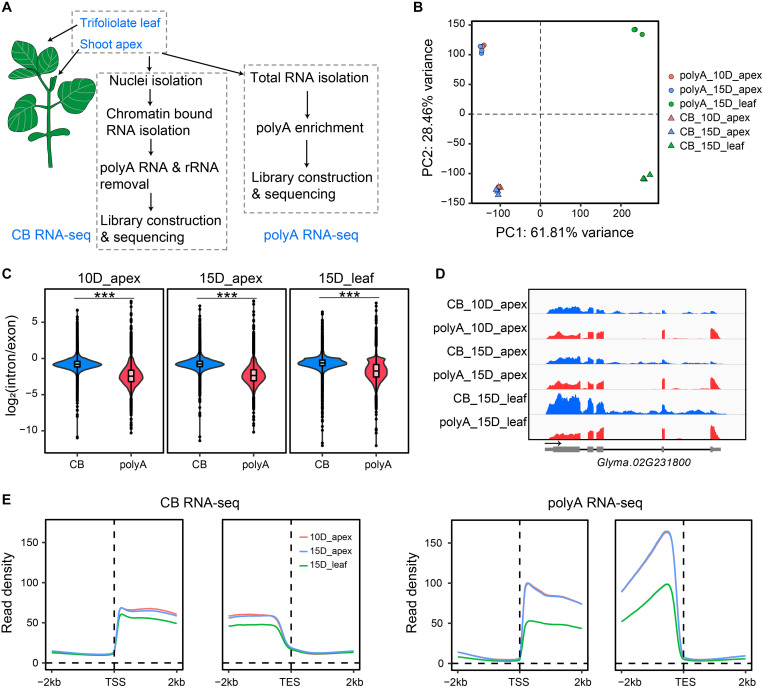
Overview of the experimental design and features of nascent RNA and mRNA. **(A)** Scheme of chromatin-bound RNA sequencing (CB RNA-seq) and polyA RNA-seq. **(B)** Principal component analysis (PCA) of gene expression of biological triplicates from CB RNA-seq and polyA RNA-seq. The triangles and dots represent CB RNA-seq and polyA RNA-seq, respectively. Red, 10-day apex; blue, 15-day apex; green, 15-day leaf. **(C)** Comparison of the gene intron/exon ratio between CB RNA-seq and polyA RNA-seq (left, 10-day apex; middle, 15-day apex; right, 15-day leaf). ***p < 0.001, Wilcoxon test. **(D)** Screenshot of IGV showing the read distribution of CB RNA-seq and polyA RNA-seq on the *Glyma.02G231800* gene. Blue, CB RNA-seq; red, polyA RNA-seq. **(E)** Profiles of read density of CB RNA-seq (left) and polyA RNA-seq (right) for the 2-kb up- and downstream transcription start site (TSS) and transcription end site (TES). Lines represent the mean value of read density. Ten-day apex, 15-day apex, and 15-day leaf samples are indicated in red, blue, and green, respectively.

The spatial and temporal expression of genes in the shoot apex largely determines the architecture of crop plants, including the numbers of branches, flowers, and nodes, which finally affect the yield per plant. Specifically, mRNA of *Dt1* was detected in the shoot apex at 15 days after emergence under a long-day condition (Liu et al., 2010); therefore, we set to investigate the transcriptome of the shoot apex from 10- to 15-day-old plants (Figure 1A, see section “Materials and Methods”). To gain insights of the shoot apex-specific gene, we chose the first trifoliolate leaves from 15-day-old plants as control. For nascent RNA, CB RNA was isolated, and the rRNA and polyA RNA it contained were depleted prior to library construction and high-throughput sequencing as described by Zhu et al. (2020). To further reveal cotranscriptional and posttranscriptional processes, we also conducted parallel mature polyA RNA-seq by enriching polyA RNA from total RNA, and these RNAs were constructed into libraries. Three biological replicates were sequenced and analyzed for each tissue. Principal component analysis (PCA) and Pearson correlation analysis of gene expression indicated high reproducibility of biological replication (Figure 1B and Supplementary Figure 1). In addition, the first two components of PCA explained more than 90% of the variation, indicating that the tissue difference (apex vs. leaf, 61.81% of variance) and methodological difference (CB RNA-seq vs. polyA RNA-seq, 28.46% of variance) were the dominant factors for intersample differentiation (Figure 1B).

As expected, the read distribution of nascent RNA shows two characteristics compared with that of polyA RNA. First, CB RNA-seq detected more intron signals than polyA RNA-seq because more unspliced reads were sequenced at the nascent RNA level. Approximately 25% of unique mapped reads were located in the intron region with CB RNA-seq, while less than 4% of unique mapped reads were located in the intron region with polyA RNA-seq (Supplementary Figure 2). In addition, the read density ratio of introns to exons in CB RNA was significantly higher than that in polyA RNA (Figure 1C). Second, the read density on the gene decreased gradually from the 5′ end to the 3′ end, while there was no such phenomenon in polyA RNA (Figures 1D,E). For example, the read signal of the gene *Glyma.02G231800* declined from 5′ to 3′ in CB RNA-seq but not in polyA RNA-seq. Furthermore, an intron signal was evident in CB RNA but absent from polyA RNA (Figure 1D). These characteristics were consistent with the results from previous studies and confirmed that the CB RNAs obtained here were bona fide transcriptional processing nascent RNAs (Li et al., 2020; Zhu et al., 2020).

### Multiple Factors Regulate Cotranscriptional Splicing Efficiency

**FIGURE 2:**
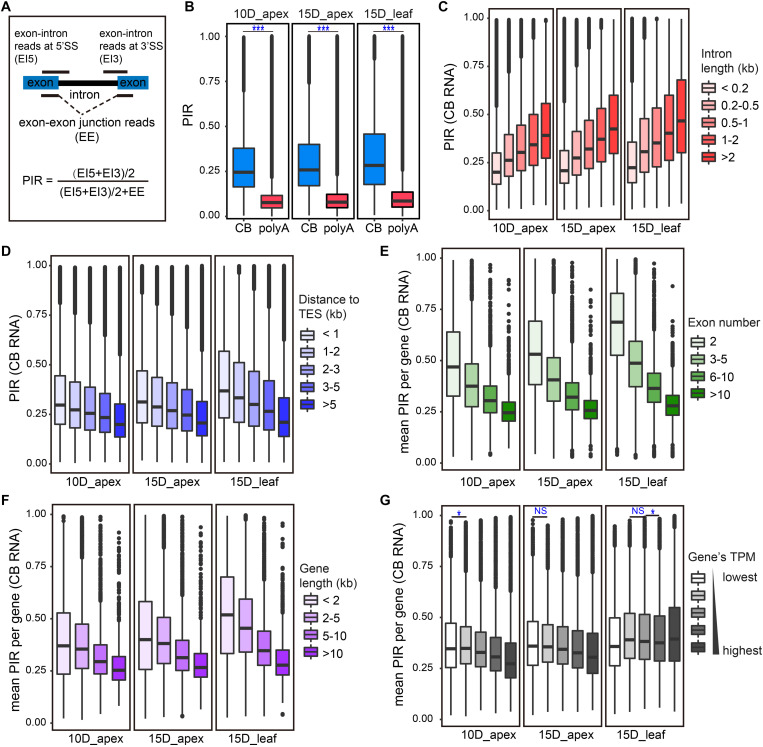
CB RNA-seq detected cotranscriptional splicing processing. **(A)** Calculation of percent of intron retention (PIR). 5′SS, 5′ splice site; 3′SS, 3′ splice site. **(B)** Boxplots of the overall PIR of CB RNA and polyA RNA for 10D_apex (left), 15D_apex (middle), and 15D_leaf (right). ***p < 0.001, Wilcoxon test. **(C,D)** Boxplots of PIR levels of introns of different sizes **(C)** and distances from the transcription end site (TES) **(D)**. **(E–G)** Boxplots of the average PIR from genes with different exon numbers **(E)**, gene lengths **(F)**, and expression levels **(G)**. For **(C–G)**, the Wilcoxon test was used to test the difference in PIRs for adjacent groups. All tests were highly significant (*p* < 0.001) unless symbols were assigned (*p < 0.05; NS, *p* > 0.05).

Cotranscriptional splicing has been widely found in eukaryotic cells. We wondered whether splicing coupled with transcription is widespread in the soybean genome. The intron retention ratio is an indicator of intron splicing efficiency. Thus, we adopted an index for the percent of intron retention (PIR) to measure the extent of cotranscriptional splicing (Braunschweig et al., 2014). In short, the PIR of an intron was calculated as the ratio of unspliced exon–intron junction reads to the total junction reads (unspliced exons–introns and spliced exons–exons). Since each unspliced exon–intron read from one RNA molecule has the chance to be sequenced twice in high-throughput sequencing, the average count of exon–intron reads at the 5′ splice site (EI5) and of exon–intron reads at the 3′ splice site (EI3) was considered an intron’s unspliced exon–intron read count (Figure 2A). Introns with lower PIR values are more efficient for splicing. Constitutive introns of active genes (TPM > 1) were calculated for PIR both in CB RNA and polyA RNA. As expected, the intron retention levels of CB RNA were significantly higher than those of polyA RNA, both in the apex and leaf (Figure 2B). Most introns in polyA RNA have a very low PIR, usually smaller than 0.1. The median PIR was close to 0.25 (in the apex) or above 0.25 (in the leaf) in CB RNA. These results were similar to those of a previous study of *Arabidopsis* (Li et al., 2020). The PIR of most introns in CB RNA was lower than 0.5 (PIR = 1 means completely unspliced), indicating the existence of genome-wide cotranscriptional splicing in soybean.

Although most introns undergo cotranscriptional splicing, the extent of intron retention is highly variable. Studies in *Drosophila* and *Arabidopsis* have indicated that multiple factors, such as intron characteristics, gene expression level, and number of introns, are related to cotranscriptional splicing efficiency (Khodor et al., 2011; Li et al., 2020; Zhu et al., 2020). To examine how these factors affect the splicing efficiency in soybean, we first divided introns into five groups by length and found that intron retention became more prominent as the intron length increased (Figure 2C).

In addition to intron length, the intron position is also supposed to influence splicing efficiency. According to the “first come, first served” model, there may be more splicing chances for introns transcribed first (Aebi et al., 1986). Based on the distance to transcription end sites (TES), introns were divided into five groups, and the PIR was compared among groups. Introns more distant from TES are transcribed early and thus are more likely to be spliced first. As expected, the PIR index gradually declined as the intron distance to TES decreased (Figure 2D).

In addition, the cotranscriptional splicing efficiency was positively correlated with exon number (Figure 2E) and gene length (Figure 2F). These patterns were consistent between the apex and leaf tissues. However, a weak positive correlation of cotranscriptional splicing and gene expression was detected in the apex instead of in the leaf (Figure 2G).

### Cotranscriptional Splicing Efficiency Is Correlated With Certain Histone Modifications

**FIGURE 3:**
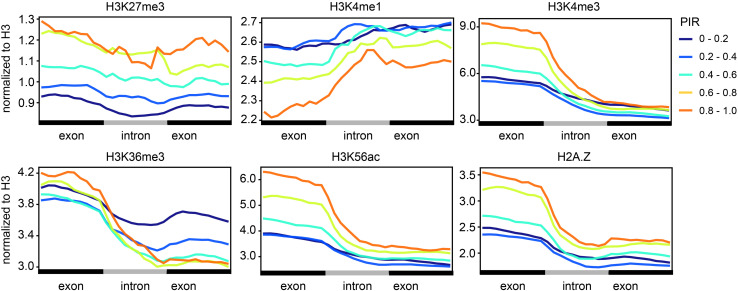
Cotranscriptional splicing efficiency is correlated with certain histone modifications. Levels (*y*-axis) of different histone modifications (H3K27me3, H3K4me1, H3K4me3, H3K36me3, H3K56ac, and H2A.Z) along introns and the flanking exons (*x*-axis). Lines with different colors indicate intron groups divided according to the PIR. The ChIP-seq data of different histone modifications in leaf tissue were adopted from a previous study (Supplementary Table 1; Lu et al., 2019).

Specific histone modifications have been shown to regulate cotranscriptional splicing by either directly recruiting spliceosomes or indirectly influencing transcriptional elongation (Luco et al., 2010; Hu et al., 2020). To test whether cotranscriptional splicing is associated with certain histone modifications in soybean, we used ChIP-seq data of several histone modifications (H3K27me3, H3K4me1, H3K4me3, H3K36me3, H3K56ac, and H2A.Z) in leaf tissue collected from a previous study (Supplementary Table 1; Lu et al., 2019). We then quantified the level of different histone modifications around introns in different groups based on the retention rates (Figure 3). PIR is positively correlated with the levels of H3K27me3, H3K4me3, H3K56ac, and H2A.Z-marked histone, which means that introns with higher cotranscriptional splicing efficiency have lower levels of those histone modifications. PIR is negatively correlated with the level of H3K4me1-marked histones. Notably, H3K27me3, H3K4me3, H3K56ac, H3K36me3, and H2A.Z showed a higher modification level at the upstream exon than at the downstream exon, while H3K4me1 showed a higher modification level at the downstream exon. It is most likely that these histone modifications, H3K27me3, H3K4me3, H3K56ac, H3K36me3, and H2A.Z, preferentially locate at the gene’s 5′ end, except for H3K4me1 (Supplementary Figure 3).

### Alternative Splicing Events Are Likely Determined Cotranscriptionally

**FIGURE 4:**
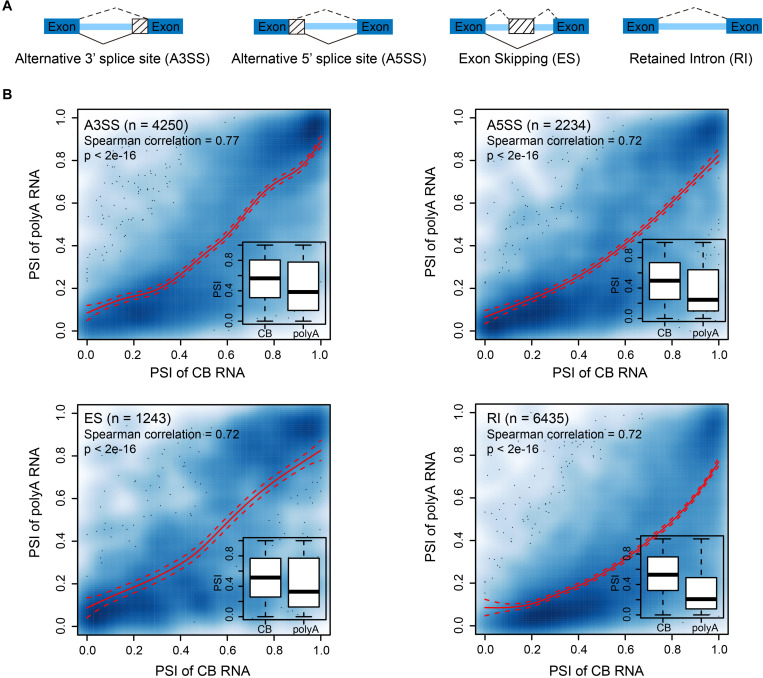
Alternative splicing events are likely determined cotranscriptionally. **(A)** Diagram showing the different alternative splicing events analyzed. **(B)** Scatter plots showing the correlation between the percent spliced-in (PSI) values of CB RNA and polyA RNA of different AS events in 15-day apex tissues. Smooth spline curves were fitted (solid red lines), and 95% confidence intervals were plotted (dashed red lines). Spearman’s correlation coefficients are presented in the plots. Insets show boxplots of PSIs for AS events at the CB RNA and polyA RNA levels.

In higher eukaryotes, alternative splicing (AS), as an important regulatory step of gene expression, plays a critical role in the development and stress response of organisms (Baralle and Giudice, 2017; Laloum et al., 2018). Previous studies in mammalian cells and *Arabidopsis* showed that AS events occur co- or post-transcriptionally (Jia et al., 2020). Thus, we wondered to what extent AS is determined cotranscriptionally. We adopted percent spliced-in (PSI) (Wang et al., 2008) to describe the relative abundance of splicing events. We focused on four AS events: alternative 3′ splice sites (A3SS), alternative 5′ splice sites (A5SS), exon skipping (ES), and retained introns (RI) (Figure 4A). The PSI values of AS events from CB RNA and polyA RNA were significantly correlated, suggesting that AS events are likely determined cotranscriptionally for all AS types (Figure 4B). This was true for both shoot apex and leaf tissues (Figure 4 and Supplementary Figure 4). However, the overall PSI value was higher in CB RNA (Figure 4B, insets). For AS events with a higher PSI in CB RNA than in polyA RNA, there are two possible explanations. First, some highly abundant transcripts in CB RNA with AS events may likely be rapidly degraded. For example, coupling of AS and nonsense-mediated mRNA decay (NMD) has been reported to fine-tune gene expression (McGlincy and Smith, 2008). Second, posttranscriptional splicing may lead to a higher PSI in CB RNA, especially for RI events.

### Differential Alternative Splicing Between Leaf and Shoot Apex Tissues Is Not Determined Merely by Cotranscriptional Splicing

**FIGURE 5:**
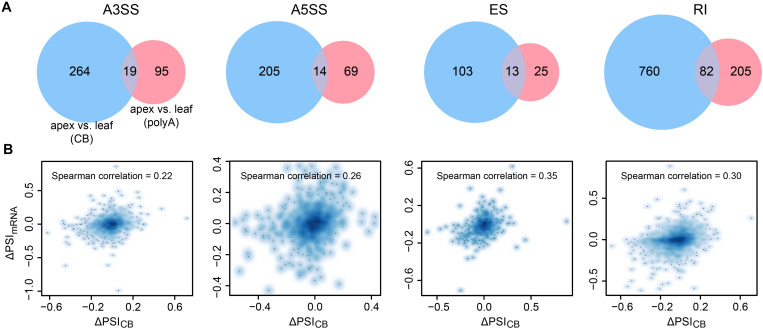
Differential AS events detected by CB RNA and polyA RNA between the 15-day apex and leaf tissues. **(A)** Venn diagram illustrating the different AS events analyzed using CB RNA and polyA RNA. **(B)** Scatter plots show the correlation of ΔPSI of CB RNA (ΔPSI_*CB*_) and polyA RNA (ΔPSI_*mRNA*_) for AS events. Spearman correlations are indicated.

Given that most AS events are determined cotranscriptionally, we then asked whether differences in AS between the shoot apex and leaf tissues detected by CB RNA-seq and polyA RNA-seq are consistent. Thus, we compared the AS difference of both CB RNA and polyA RNA between the 15-day apex and leaf tissues. Differential splicing events were analyzed by the program SUPPA2 (Trincado et al., 2018). A splicing event was considered differential when the absolute value of the PSI difference (ΔPSI) between tissues >0.1 and the *p-*value < 0.05. A small number of the different splicing events between the leaf and shoot apex tissues were detected by both CB RNA and polyA RNA (Figure 5A). ΔPSI_*mRNA*_ and ΔPSI_*CB*_ were barely correlated (Spearman correlation ranged from 0.22 to 0.35) (Figure 5B). Furthermore, genes with different splicing events detected by CB RNA and polyA RNA were not concordant (Supplementary Figure 5A). Although overall AS events are highly correlated at the cotranscriptional level and posttranscriptional level within the same tissue, tissue-specific mRNA processing, such as degradation and posttranscriptional splicing, may result in the differential AS events that are detected by polyA RNA but not by CB RNA. For those differential AS events detected by CB RNA but not by polyA RNA, it was probably caused by the differentially cotranscriptional splicing efficiency between the shoot apex and leaf tissues and further corrected at the posttranscriptional splicing step, exemplified by the first intron of *Glyma.07G206100* (Supplementary Figure 6).

Genes associated with intertissue differential splicing events detected by CB RNA and polyA RNA were also different (Supplementary Figure 5A). To explore the biological function of genes with different AS events, we conducted Gene Ontology (GO) enrichment analysis. Interestingly, genes with different splicing events between the 15-day apex and leaf tissues were significantly enriched in mRNA splicing and RNA processing, which somehow explains the differential splicing efficiency between the shoot apex and leaf tissues (Supplementary Figure 5B).

### The Level of Steady-State mRNA Is Moderately Correlated With the Biogenesis of Nascent RNA

**FIGURE 6:**
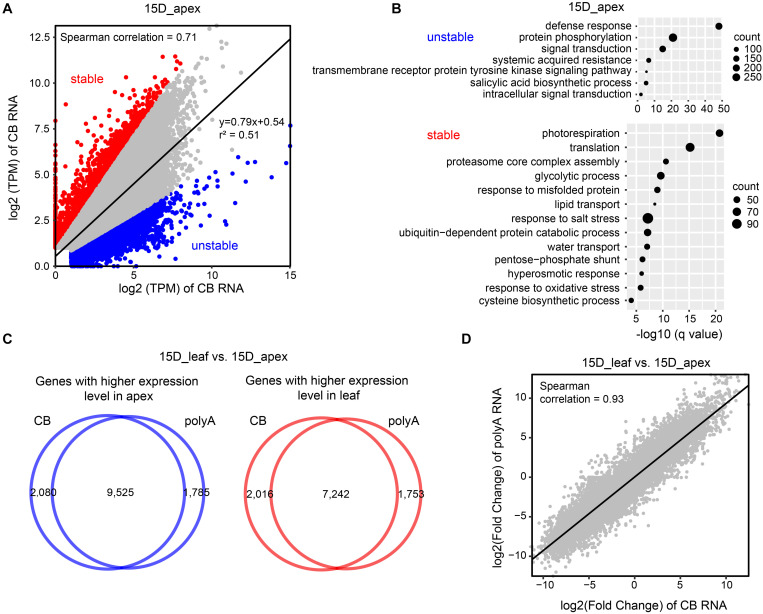
Comparison of the expression levels of CB RNA and polyA RNA. **(A)** Scatter plot of gene TPM values (in log2) detected by CB RNA-seq and polyA RNA-seq in a 15-day apex. Spearman correlation and linear regression (solid line) equations are shown. Stable and unstable transcripts are represented by red and blue dots, respectively. **(B)** Gene Ontology (GO) enrichment of genes with unstable (upper) and stable RNA (bottom). **(C)** Venn diagrams show the number of genes with higher expression levels in 15-day apexes (left) and 15-day leaves (right) at the CB RNA and polyA RNA levels. These genes were detected by comparing DEGs between 15-day apex and 15-day leaf tissues (| fold change| > 2, *q*-value < 0.05). **(D)** Scatter plot of fold change of gene expression (15-day apex vs. 15-day leaf) detected by CB RNA-seq and polyA RNA-seq. The Spearman correlation value is presented.

Chromatin-bound RNA-seq is applied to detect transcribed RNAs, which are subject to multiple steps of mRNA processing, including cotranscriptional and posttranscriptional processes prior to maturation. Thus, there might be discordance in the abundance at the nascent RNA and mRNA levels. To test this hypothesis, we compared the TPM values of nascent RNA and mature RNA. Overall, the levels of nascent RNA and mature RNA were moderately correlated (Spearman correlation = 0.71–0.73) (Figure 6A and Supplementary Figures 7A–C). There are two types of discordant genes. One is a gene that is highly transcribed with a low level of mature RNA, which might result from a high turnover of mRNA and is designated unstable RNA. The other is a gene with relatively low transcription activity but a high level of mature RNA, which might be due to the high RNA stability and is called stable RNA.

To select unstable and stable RNA transcripts, we first established a linear regression model of the log2 values of TPM genes obtained with CB RNA-seq and polyA RNA-seq. Then, the predicted TPM values of genes in polyA RNA were calculated based on the linear regression model. If the actual TPM of a gene was threefold higher (or lower) than the predicted TPM, the gene was considered to be stable (or unstable) (Figure 6A and Supplementary Figures 7B,C). To investigate whether the stability of RNA is associated with specific biological functions, we performed GO enrichment analysis. For unstable RNAs, defense response, protein phosphorylation, and signal transduction were the most enriched terms. Stable RNAs were mainly associated with translation, photorespiration, ribosome biogenesis, and glycolytic processes (Figure 6B and Supplementary Figures 7C,E).

### Differentially Expressed Genes Are Consistent at the Nascent and Mature RNA Levels

We then identified differentially expressed genes (DEGs) between 15-day apex and 15-day leaf tissues at both nascent and mature RNA levels. More than 10,000 genes were expressed more in the apex than in the leaf, and vice versa (Supplementary Figure 8A and Supplementary Table 2). Most of these DEGs detected by CB RNA-seq and polyA RNA-seq overlapped (Figure 6C and Supplementary Figure 8B). Furthermore, fold changes at the CB RNA level and polyA RNA level were highly correlated (Spearman correlation = 0.93) (Figure 6D).

Gene Ontology enrichment analysis was performed to determine the biological functions of the DEGs. Genes with higher expression in the apex were mainly associated with RNA methylation, histone methylation, translation, DNA replication, and meristem initiation and maintenance. Genes with higher expression levels in the leaves were mainly related to photosynthesis and plastid organization (Supplementary Figure 8C).

In addition, only a small number of genes were called DEGs between the 15-day apex and 10-day apex (Supplementary Figure 9A), and they had concordant changes at the nascent RNA and mRNA levels (Supplementary Figure 9B). GO enrichment indicated that genes highly expressed in the 10-day apex were involved in the response to stress, circadian rhythm, etc., and genes highly expressed in the 15-day apex were involved in long-day photoperiodism flowering, response to hormones, and circadian rhythm (Supplementary Figure 9C).

### More Non-coding RNAs Were Identified by CB RNA-Seq Than PolyA RNA-Seq

**FIGURE 7:**
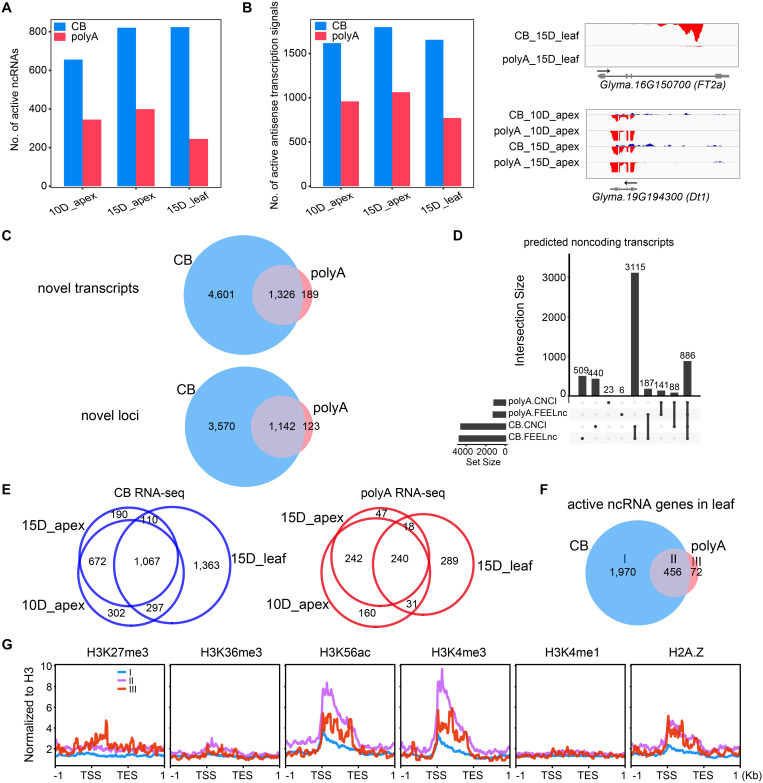
CB RNA-seq detected more ncRNA transcripts than polyA RNA-seq. **(A)** Number of active ncRNAs detected by CB RNA-seq and polyA RNA-seq. **(B)** Number of active antisense transcription signals detected by CB RNA-seq and polyA RNA-seq (left). Two examples of ncRNAs are shown in the IGV screenshot (right). **(C)** Venn diagrams show the novel transcripts (upper) or the related loci (bottom) detected by CB RNA-seq and polyA RNA-seq. **(D)** UpSet plot shows the number of ncRNAs defined by CNCI and FEELnc in CB RNA and polyA RNA. **(E)** Venn diagrams show the overlapping ncRNA genes of the shoot apex and leaf tissues at the CB RNA (left) and polyA RNA levels (right). **(F)** Venn diagram showing three types of active ncRNAs in the leaves. Group I, only detected by CB RNA; group II, detected by both; group III, only detected by polyA RNA. **(G)** The average distribution of different histone modifications in each ncRNA group.

Considering that unstable transcripts are readily detected at the nascent RNA level, we calculated the expression level of ncRNA as defined in a previous study (Lin et al., 2020). As expected, more active ncRNA genes were detected by CB RNA-seq than polyA RNA-seq (Figure 7A). Furthermore, we determined the antisense transcription of annotated mRNAs by counting reads mapped to the opposite strand, and there were more active antisense transcriptional signals at the nascent RNA level (Figure 7B, left). These results indicate that some non-coding transcripts were unstable or not polyadenylated. For example, a transcript encoded from the antisense strand of *FT2a*, the essential gene involved in flowering timing, was identified in 15-day leaves by CB RNA-seq. *Dt1*, the key gene controlling growth habit, overlapped with another strong antisense transcript at the nascent RNA level in the apex (Figure 7B, right).

To identify novel transcripts, we assembled transcripts from nascent RNA and polyA RNA of each tissue separately. Then, all transcripts were merged and compared based on reference annotations (see section “Materials and Methods”). Only intergenic transcripts were included for further analysis. In total, there were 5,927 and 1,515 active intergenic transcripts from CB RNA-seq and polyA RNA-seq, respectively, with 1,326 transcripts overlapping (Figure 7C, upper panel; Supplementary Table 3). These transcripts were encoded from 4,835 loci, of which 1,142 were shared by CB RNA and polyA RNA (Figure 7C, bottom panel).

We then applied two tools, CNCI and FEELnc, to evaluate the protein-coding potential of these new transcripts. In total, 4,001 and 974 active new transcripts of CB RNA and polyA RNA were considered non-coding transcripts by both methods, respectively (Figure 7D), and more ncRNAs were observed in the leaves at the nascent RNA level (Figure 7E).

Non-coding RNA detected only at the nascent RNA level might be unstable or unpolyadenylated. ncRNAs detected only at the polyA RNA level might be very stable and accumulate by slow transcription. Different types of ncRNAs may be regulated differently at the transcriptional level. To gain insight into the effects of histone modifications on ncRNA expression, we compared the metaprofiles of histone modifications for three groups of ncRNAs from the leaf tissue (group I: only detected by CB RNA; group II: detected by both; group III: only detected by polyA RNA) (Figure 7F). Group II and III ncRNA genes were associated with H3K56ac, H3K4me3, and histone variant H2A.Z (Figure 7G).

## Discussion

Although nascent RNA-seq has been extensively used to detect cotranscriptional regulation in yeast, fly, and mammalian cells, its application in plants is still lagging behind. Recently, several methods have been developed to detect nascent RNA and reveal plant-specific transcriptional features (Hetzel et al., 2016; Zhu et al., 2018). However, with the exception of one maize publication using GRO-seq (Erhard et al., 2015), all studies have focused on the model plant *Arabidopsis*. Here, we describe the soybean transcriptome using CB RNA-seq. As expected, CB RNA isolation greatly enriched the nascent RNA by removing the abundant cytosolic mRNAs and nucleoplasmic RNAs. We demonstrated that CB RNA-seq successfully detected nascent RNA biogenesis and cotranscriptional processing of pre-mRNA from the leaves and growing apex tissues. This method can be applied to other tissues at various developmental stages and/or under different environmental conditions, which may further shed light on the transcriptional regulation of the soybean genome.

We found genome-wide cotranscriptional splicing in soybean. Cotranscriptional splicing efficiency is related to intron length, distance from TES, intron number, and gene length. These characteristics are similar to those previously observed in yeast, fly, mammalian, and *Arabidopsis* cells, indicating a conserved mechanism that controls cotranscriptional splicing in eukaryotic cells (Khodor et al., 2011; Kindgren et al., 2020; Li et al., 2020). Interestingly, we found that both active (H3K4me3 and H3K56ac) and inactive (H3K27me3) histone markers are negatively related to cotranscriptional splicing efficiency. The elongation rate of RNA Pol II can affect splicing efficiency by fine-tuning the timing of the spliceosome search for splice sites, as the spliceosome is physically recruited by the carboxyl terminal domain of the largest subunit of RNA Pol II (Nojima et al., 2018). The inverse correlation between elongation speed and splicing efficiency was proven in yeast *in vivo* (Carrillo Oesterreich et al., 2016; Aslanzadeh et al., 2018). Moreover, the RNA Pol II elongation rate is regulated by transcription elongation factors and chromatin structural barriers such as nucleosomes. Thus, factors that affect transcription elongation also affect splicing efficiency. Active histone markers are thought to be related to a higher transcription elongation rate. Therefore, it is reasonable that introns with higher H3K4me3 or H3K56ac contents are less efficiently spliced. In addition, the pattern described in this study and a previous study on *Arabidopsis* revealed that the retained introns are derived from genes with low H3K4me1 and high H3K27me3 signatures (Mahrez et al., 2016). However, further studies of mutants with impaired histone modification are needed to verify their function in cotranscriptional splicing. Actually, these effects are not unidirectional. Cotranscriptional splicing can in turn influence the elongation rate and establishment of histone modifications (Kim et al., 2011).

Alternative splicing is an important part of gene regulation. In our study, a highly correlated relative AS event (PSI) was observed between CB RNA and polyA RNA, suggesting that most AS events are determined cotranscriptionally. This agrees with a previous study in *Arabidopsis* (Zhu et al., 2020). However, when comparing intertissue AS events, differential AS events detected at the cotranscriptional and posttranscriptional levels only partially overlapped. Thus, differential AS events cannot be predicted at the nascent RNA level, indicating the complexity of AS regulation. These regulations may be attributed to different degradation rates and/or posttranscriptional splicing among various tissues.

Gene expression is regulated at multiple levels, including transcription, post-transcription, and translation. Steady-state mRNA is the output of transcriptional activity and RNA degradation. Thus, there might be some discordance in gene activity detected by nascent RNA-seq and polyA RNA-seq. As expected, we found that gene activity at these two levels was moderately correlated. However, when comparing different tissues, the changes in gene activity at both levels were highly consistent, indicating that tissue-specific gene expression was mainly associated with transcription. The stability of RNA might contribute to the discordance in gene activity at the nascent and mature RNA levels. It is meaningful for stable mRNA genes to be involved in housekeeping biological processes. Moreover, under normal conditions, keeping regulatory genes at low mRNA levels and relatively high transcription by fast turnover of mRNA is an effective way to ensure rapid responses to potential stimuli. As we have previously reported in *Arabidopsis*, genes induced highly and quickly by short-term heat shock usually exhibit basic transcription under normal temperature (Liu M. et al., 2020).

Since some ncRNAs are unstable or unpolyadenylated, such as enhancer RNAs and antisense RNAs, more transcripts are expected to be detected by CB RNA-seq. However, this does not rule out the possibility that some transcripts detected only in CB RNA are not nascent RNA but rather chromatin-bound transcripts. To further elucidate the biological significance of these ncRNAs, approaches such as RNA interference and gene editing are needed. It will be interesting to apply CB RNA-seq to various tissues and build a transcriptional regulatory network at the nascent RNA level in the future.

## Materials and Methods

### Plant Materials and Growth Conditions

Soybean Wm82 plants were grown under long light day conditions (16 h light, 8 h dark) with a constant 25°C temperature in a growth chamber. Shoot apexes from 10- to 15-day seedlings were collected in three biological replicates, with each replicate collected from approximately 20 plants. For the leaves, the first trifoliolate leaves of two 15-day-old plants were collected as one biological replicate. All samples were frozen with liquid nitrogen immediately after collection.

## Rna Isolation, Transcriptome Library Preparation, and Sequencing

The chromatin RNA extraction protocol was modified from a previously published method (Zhu et al., 2020). Briefly, tissues were ground into a fine powder with liquid nitrogen and solubilized in cold nuclei isolation buffer (20 mM/KOH pH 7.4, 0.44 M sucrose, 1.25% Ficoll, 2.5% Dextran T40, 10 mM MgCl_2_, 0.75% Triton X-100, 0.5 mM EDTA, 1 mM DTT, 8 mM β-mercaptoethanol, 1 μg/ml pepstatin A, 1 μg/ml aprotinin, and 1 mM PMSF). The crude nuclei were precipitated at 3,500 rpm and washed with resuspension buffer (50% glycerol, 25 mM Tris–HCl pH 7.5, 0.5 mM EDTA, 100 mM NaCl, 1 mM DTT, 0.4 U/μl RNase inhibitor, 1 μg/ml pepstatin A, 1 μg/ml aprotinin, and 8 mM β-mercaptoethanol) once, followed by washing buffer (25 mM Tris–HCl pH 7.5, 300 mM NaCl, 1 M urea, 0.5 mM EDTA, 1 mM DTT, 1% Tween-20, 0.4 U/μl RNase inhibitor, 1 μg/ml pepstatin A, 1 μg/ml aprotinin, and 8 mM β-mercaptoethanol) twice. Chromatin RNA was extracted from washed nuclei using TRIzol reagent (Life Technologies).

After degrading genomic DNA by TURBO DNase (Life Technologies), CB RNA was subjected to rRNA depletion using a riboPOOL kit (siTOOLs Biotech, PanPlant-10 nmol) and polyA RNA removal by oligo(dT) beads (NEB, S1419). Poly(A) RNA was enriched from total RNA by oligo(dT) beads. Both CB RNA and polyA RNA were transformed into cDNA libraries using the NEBNext Ultra II Directional RNA Library Prep Kit for Illumina (NEB #E7765) and sequenced on an Illumina NovaSeq platform.

### CB RNA and mRNA Data Processing

Raw reads of CB RNA and polyA RNA were first evaluated by FastQC^1^, and then Cutadapt was used to remove adapters and low-quality reads (Martin, 2011). Clean reads were subsequently aligned to the genome Wm82.a2.v1 by STAR (Dobin et al., 2013). Only uniquely mapped reads were retained for the following analysis. Read distribution on genomic features was evaluated by RSeQC with the subcommand “read_distribution.py” (Wang et al., 2012). To calculate the ratio of introns vs. exons of each gene, featureCounts was used to quantify the read counts on introns and exons separately (Liao et al., 2014). Read density was normalized by the length of introns and exons.

### Calculating the Percent of Intron Retention

The proportion of intron-retained reads across an intron is usually used to evaluate the splicing efficiency of the intron. To quantitatively evaluate the genome-wide cotranscriptional splicing efficiency in soybean, we calculated the PIR value for constitutive introns as described previously (Braunschweig et al., 2014). Briefly, three types of reads on an intron were counted: (1) exon–intron junction reads across the 5′SS (EI5), (2) exon–intron junction reads across the 3′SS (EI3), and (3) spliced exon–exon junction reads (EE) (Figure 2A). The PIR of an intron was calculated by dividing the intron-retained reads by the sum of intron-retained reads and intron-skipping reads (Figure 2A). Constitutive introns from the annotation Wm82.a2.v1 were subjected to PIR calculations.

### Alternative Splicing Analysis

Mapped reads were assembled into putative transcripts based on a reference guided assembly strategy using the single-sample transcript assembly tool StringTie v2.1.2 (Pertea et al., 2015). Multiple putative transcripts were merged into a unified set of transcripts using the meta-assembly tool TACO v0.7.3, which was considered to be superior to Cuffmerge and StringTie merge (Niknafs et al., 2017). Then, the merged transcripts were compared with the reference gene GTF file using GffCompare v0.11.2 (Pertea and Pertea, 2020). Since CB RNA was nascent RNA with no full splicing, AS analysis was based on transcripts merged from polyA RNA data. AS events were quantified based on the PSI in the program SUPPA2 (Trincado et al., 2018). Since SUPPA2 estimated the PSI based on transcript abundance, we first used salmon for alignment-free transcript abundance estimates (Patro et al., 2017). Transcripts with TPM > 1 in at least three samples were used for analysis. For detection of differential splicing between two samples, we chose ΔPSI > 0.1 and *p*-value < 0.05 as cut-offs.

### Detection of Differentially Expressed Genes

For detecting genes with differential expression, mapped reads in each gene were quantified using featureCounts. Then, differential gene expression was evaluated by the R package DESeq2 (Love et al., 2014). DEGs were defined by the following criteria: they had to show more than twofold up- or downregulation, and the false discovery rate (FDR)-adjusted *q*-value calculated by DESeq2 had to be less than 0.05. The read density for each gene was calculated by normalizing the read count to the library size and mappable length (TPM).

### Gene Ontology Enrichment Analysis

Gene Ontology annotation of genes was extracted from the annotation file for Wm82.a2.v1. A hypergeometric test was explored for the statistical test, and the Benjamini and Hochberg method (1995) was used to adjust the *p*-value to control the FDR. All analysis was done in R software.

### Detection of New Non-coding RNA Genes

To detect new ncRNA genes at the nascent RNA and polyA RNA levels, transcripts were assembled in CB RNA and polyA RNA data separately and merged by TACO as described above in the AS event analysis. Then, annotation GTF files of transcripts were compared with reference annotation GTF files using GffCompare (with the -r option). For each putative transcript, its relationship to the closest reference transcript was described by a “class code” value. For example, the code “=” indicates that the introns of a transcript completely match the introns of the reference transcript. We chose only unknown, intergenic transcripts that were assigned the code “u” and estimated their protein-coding potential by two software programs, CNCI and FEELnc (Sun et al., 2013; Wucher et al., 2017).

### Reanalysis of ChIP-Seq Data of Histone Modifications

ChIP-seq raw data of histone modifications were downloaded from NCBI (Supplementary Table 1). The raw data were first processed with adapter removal by Cutadapt and mapping to the genome by STAR. Then, the average distribution of different histone modifications on genomic features was plotted using deepTools by normalization to histone 3 (Ramírez et al., 2016).

## Data Availability Statement

The original contributions presented in the study are publicly available. This data can be found here: NCBI website under Bioproject accession PRJNA689321.

## Author Contributions

ZD, ML, and JZ designed the research. JZ and ML performed the research. ML, HZ, FK, and BL analyzed the data. ML and ZD wrote the manuscript. All authors contributed to the article and approved the submitted version.

## Conflict of Interest

The authors declare that the research was conducted in the absence of any commercial or financial relationships that could be construed as a potential conflict of interest.

## Footnotes

1 http://www.bioinformatics.babraham.ac.uk/projects/fastqc/
