# Supplementary material for: Cotranscriptional and Posttranscriptional Features of the Transcriptome in Soybean Shoot Apex and Leaf
Source: Front Plant Sci. 2021 Apr 9;12:649634. doi: 10.3389/fpls.2021.649634 (PMC8063115; doi:10.3389/fpls.2021.649634)
Supplement: Supplementary file 2 [file Data_Sheet_2.docx]

## Supplementary Figures

**Figure S1.** Hierarchical clustering with Pearson correlation of gene expression for biological triplicates of CB RNA-seq and polyA RNA-seq.

**Figure S2.** Reads distribution on genomic elements of CB RNA-seq and polyA RNA-seq.


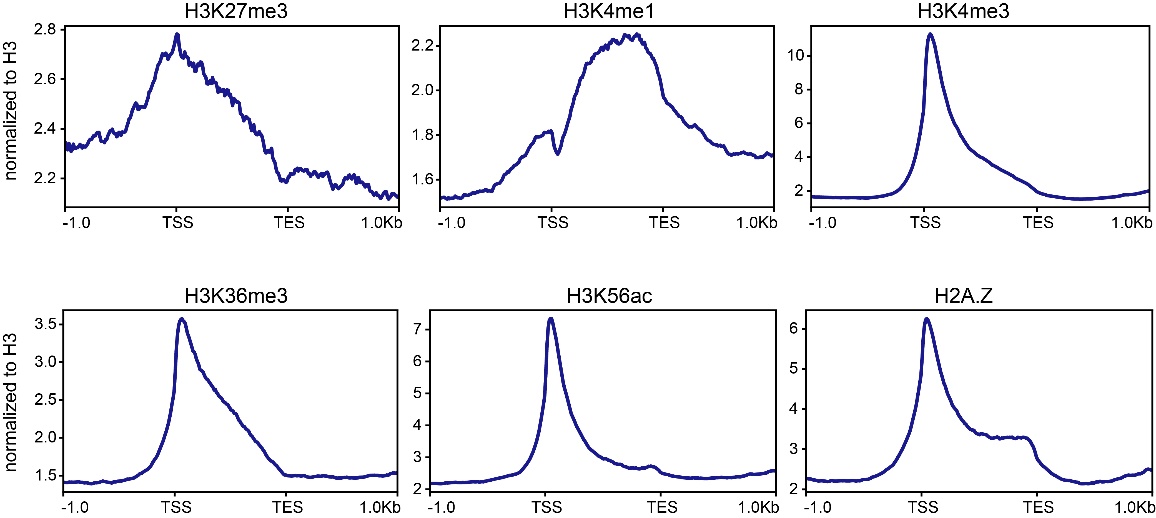


**Figure S3.** Profiles of read density (normalized to Histone 3) of histone modifications for the 1kb up- and downstream transcription start site (TSS) and transcription end site (TES). Lines represent the mean value of read density.

**Figure S4.** Scatter plots showing the correlation between PSIs of CB RNA and polyA RNA for different AS events in 10-day apex (A) and 15-day leaf (B). Smooth spline curves were fitted (solid red lines), and 95% confidence intervals were plotted (dashed red lines). Spearman’s correlation is presented in the plots.

**Figure S5.** Genes associated with differential AS events in CB RNA and polyA RNA. (A) Venn diagrams of genes associated with differential AS events by comparing 15-day apex and 10-day apex (left), 15-day leaf and 15-day apex (right). (B) GO enrichment of genes with differential AS events detected by CB RNA (left) and polyA RNA (right) in 15-day leaf vs. 15-day apex.


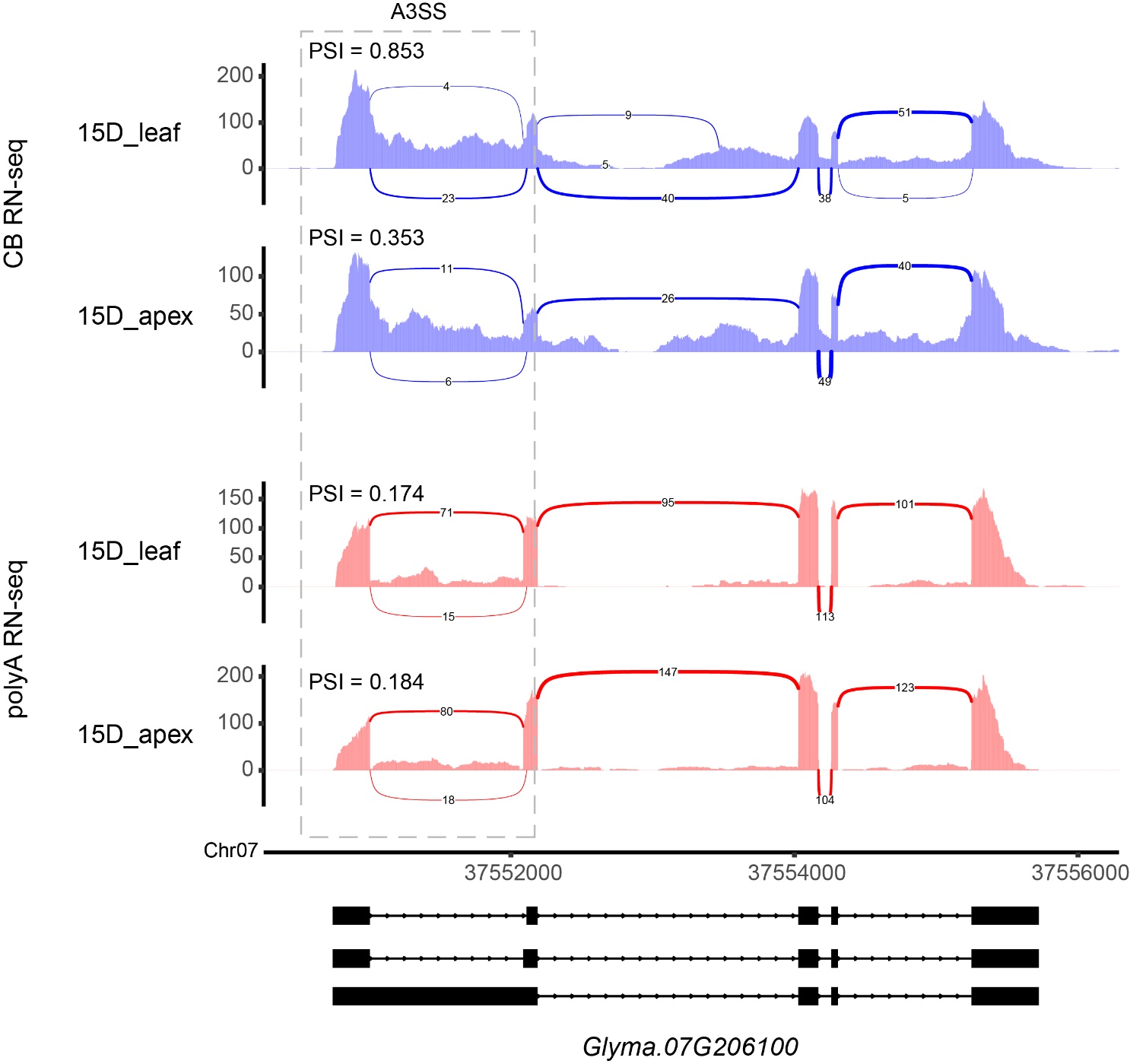


**Figure S6.** An example of alternative splicing detected only in CB RNA-seq between 15-day leaf and 15-day apex. Sashimi plots depicting splice alteration of the gene *Glyma.07G206100* in CB RNA (blue) and polyA RNA (red). The number of reads splits across the splice junction are presented on the arc lines. PSI values for the A3SS event in the first exon-exon junction were indicated.


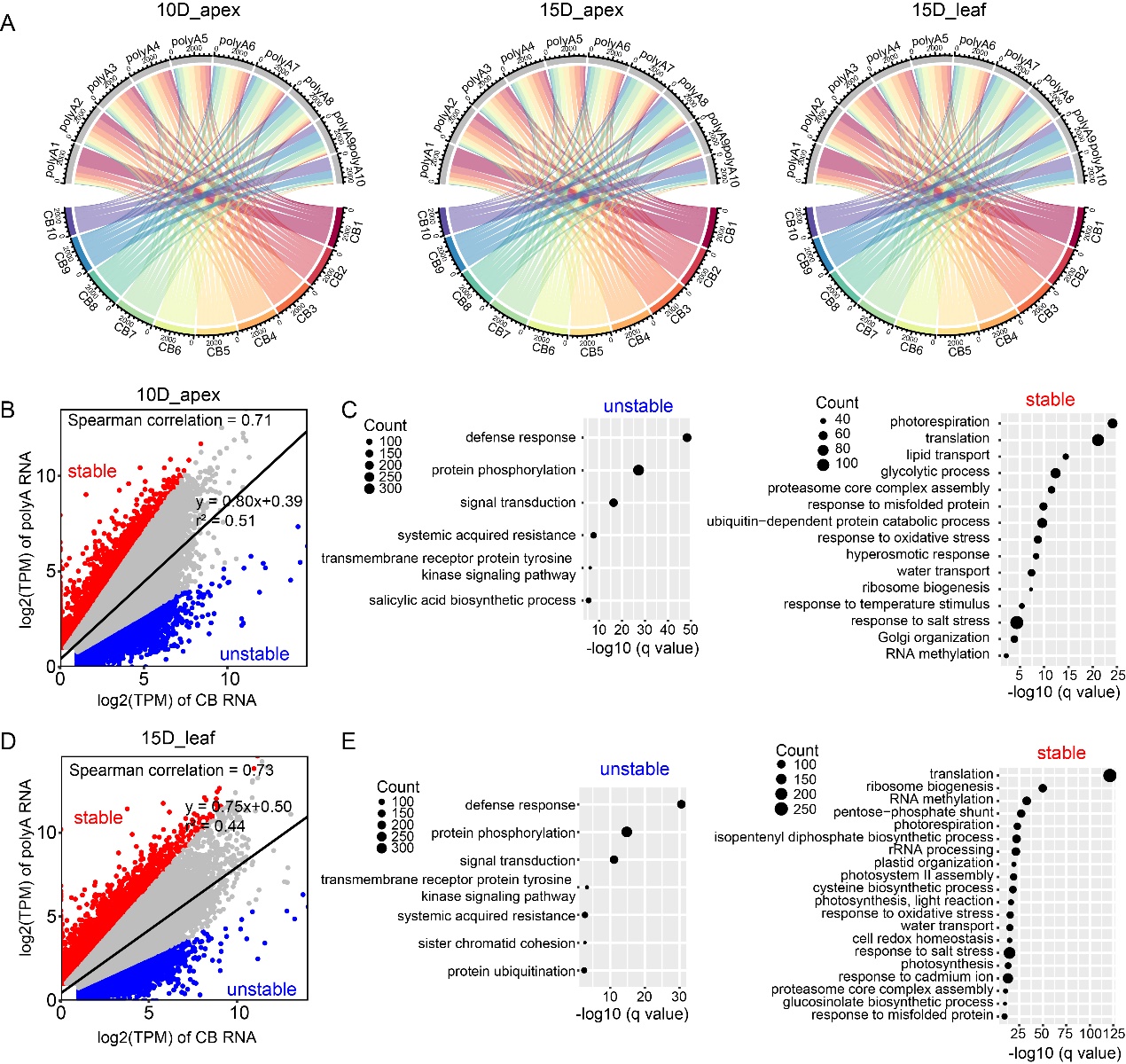


**Figure S7.** Comparison of gene expression at CB RNA and polyA RNA levels. (A) Chord diagrams showing intercorrelation of gene expression of CB and polyA RNA in 10-day apex (left), 15-day apex (middle) and 15-day leaf (right). Active genes were divided into decile groups based on expression level, descending from group 1 (CB1 or polyA1) to group 10 (CB10 or polyA10). (B) Correlation of TPM between CB RNA and polyA RNA in 10-day apex. Spearman correlation is presented. Genes with stable and unstable RNA are in red dots and blue dots, respectively. (C) GO enrichment of genes with unstable (left) and stable (right) RNA in 10-day apex. (D) Correlation of TPM between CB RNA and polyA RNA in 15-day leaf. Spearman correlation is presented. Genes with stable and unstable RNA are in red dots and blue dots respectively. (E) GO enrichment of genes with unstable (left) and stable (right) RNA in 15-day leaf.


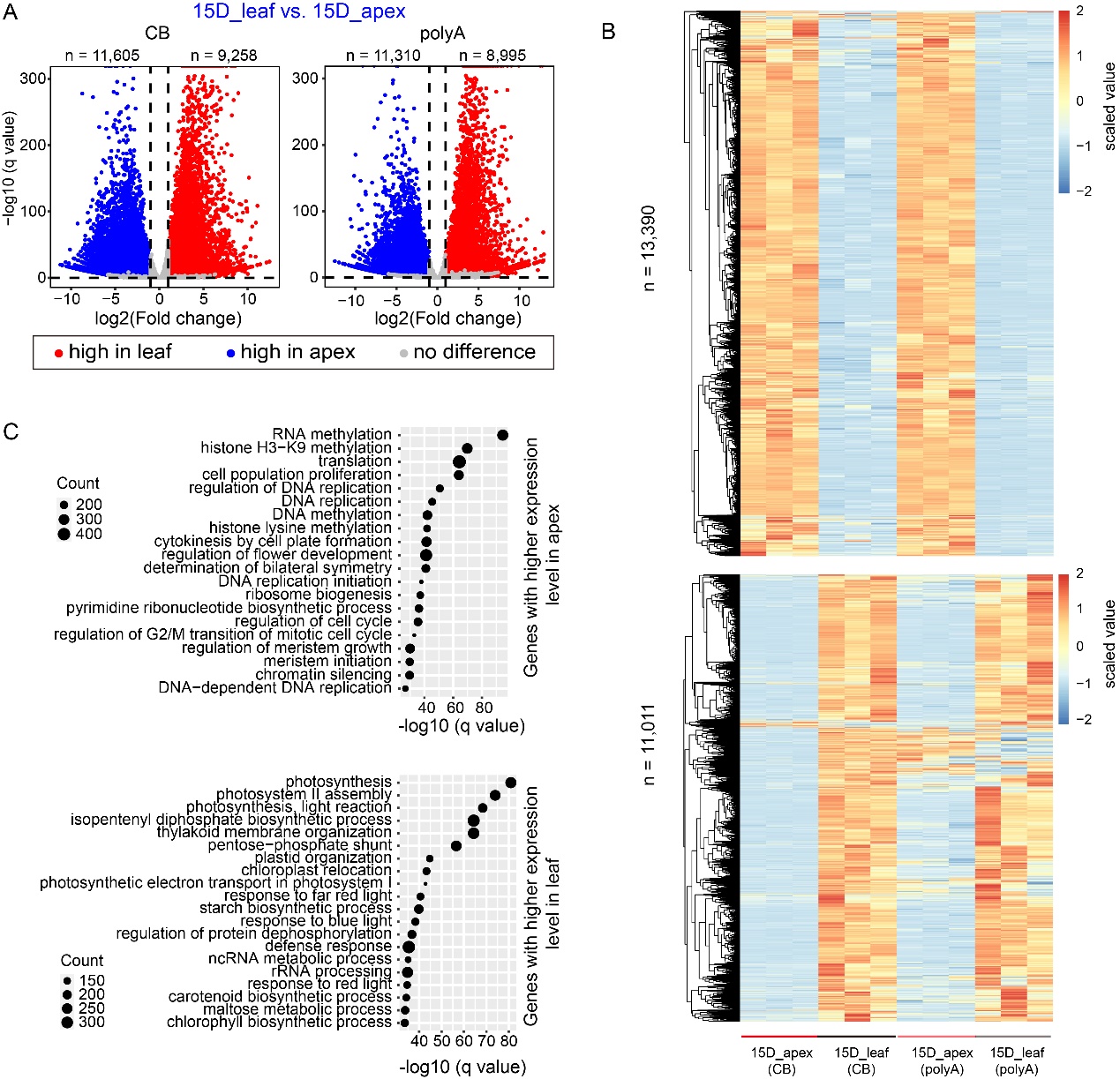


**Figure S8.** DEGs detected in 15-day leaf vs. 15-day apex. (A) Volcano plots showing the number of DEGs detected by CB RNA-seq (left) and polyA RNA-seq (right). Blue and red dots represented genes with higher expression level in apex and leaf, respectively. (B) Heatmap of the union DEG sets detected by CB RNA and polyA RNA in 15-day leaf vs. 15-day apex. The upper panel shows genes with higher expression in apex. The bottom panel shows genes with higher expression in leaf. Each line represents a gene and its TPM was scaled by row for CB RNA and polyA RNA, respectively. (C) GO enrichment of genes highly expressed in 15-day apex vs. 15-day leaf (upper) and genes highly expressed in 15-day leaf vs. 15-day apex (bottom).


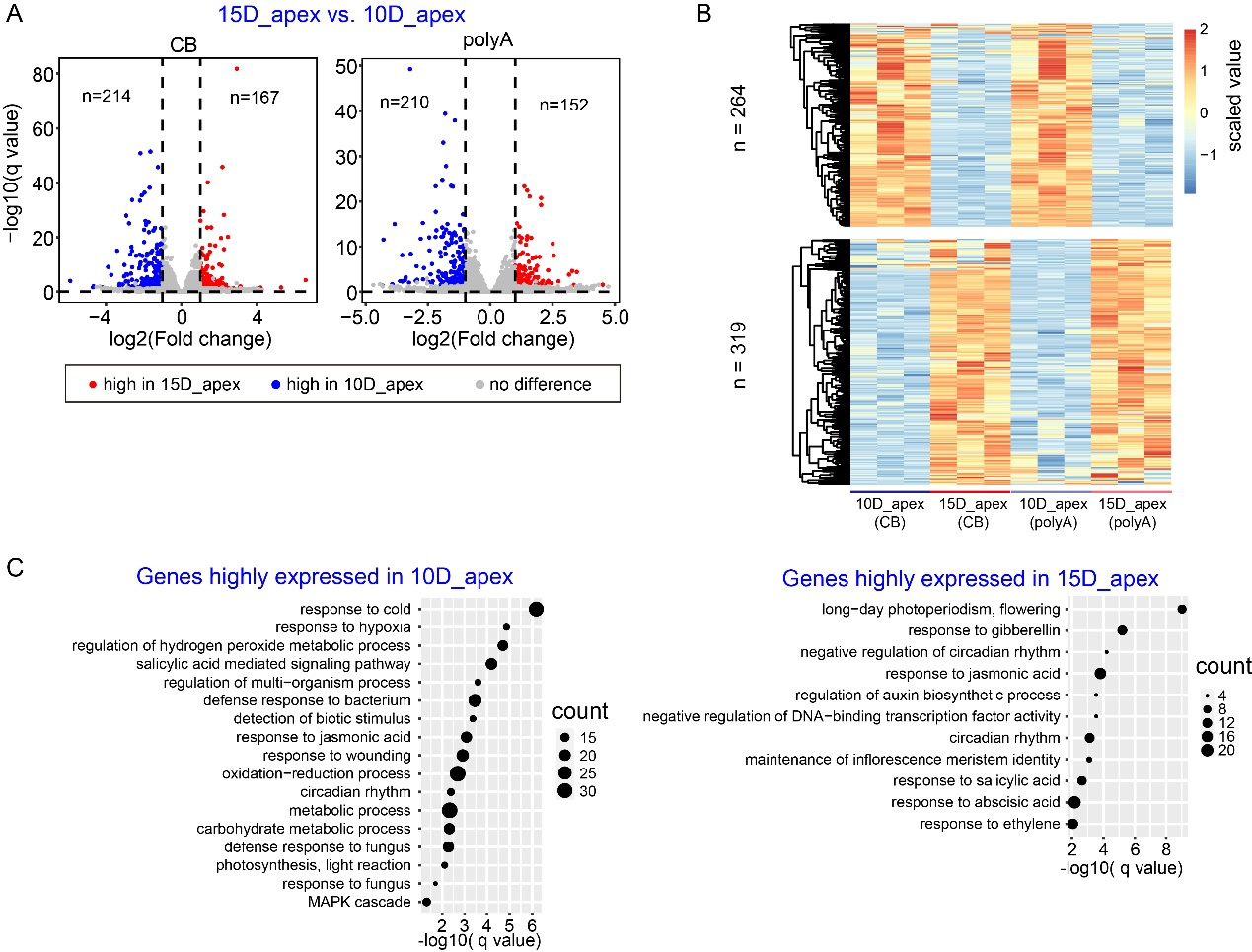


**Figure S9.** DEGs detected in 15-day apex vs. 10-day apex. (A) Volcano plots showing the number of DEGs detected by CB RNA-seq (left) and polyA RNA-seq (right). Blue and red dots represented genes with higher expression level in 10-day apex and 15-day apex, respectively. (B) Heatmap of the union DEG sets detected by CB RNA and polyA RNA in 15-day apex vs. 10-day apex. The upper panel shows genes with higher expression in 10-day apex. The bottom panel shows genes with higher expression in 15-day apex. Each line represents a gene and its TPM was scaled by row for CB RNA and polyA RNA, respectively. (C) GO enrichment of genes highly expressed in 10-day apex vs. 15-day apex (left) and genes highly expressed in 15-day apex vs. 10-day apex (right).
